# The Impact of the Microbiota on the Immune Response Modulation in Colorectal Cancer

**DOI:** 10.3390/biom15071005

**Published:** 2025-07-14

**Authors:** Ana Iulia Neagu, Marinela Bostan, Vlad Alexandru Ionescu, Gina Gheorghe, Camelia Mia Hotnog, Viviana Roman, Mirela Mihaila, Simona Isabelle Stoica, Camelia Cristina Diaconu, Carmen Cristina Diaconu, Simona Maria Ruta, Coralia Bleotu

**Affiliations:** 1 Faculty of Medicine, University of Medicine and Pharmacy Carol Davila Bucharest, 050474 Bucharest, Romania; ana-iulia.neagu@umfcd.ro (A.I.N.); drgheorghe.gina@gmail.com (G.G.);; 2 Department of Cellular and Molecular Pathology, Stefan S. Nicolau Institute of Virology, Romanian Academy, 030304 Bucharest, Romania; carmen.diaconu@virology.ro (C.C.D.); coralia.bleotu@virology.ro (C.B.); 3 Center of Immunology, Stefan S. Nicolau Institute of Virology, Romanian Academy, 030304 Bucharest, Romania; viviana.roman@virology.ro (V.R.); mirela.mihaila@virology.ro (M.M.); 4 Department of Immunology, ‘Victor Babes’ National Institute of Pathology, 050096 Bucharest, Romania; 5 Internal Medicine Department, Clinical Emergency Hospital of Bucharest, 105402 Bucharest, Romania; 6 Department of Biochemistry and Biophysics, Faculty of Midwives and Nursing, University of Medicine and Pharmacy Carol Davila Bucharest, 050474 Bucharest, Romania; 7 Faculty of Pharmacy, Titu Maiorescu University, 040314 Bucharest, Romania; 8 Academy of Romanian Scientists, 050085 Bucharest, Romania; 9 Department of Emerging Viral Diseases, Stefan S. Nicolau Institute of Virology, Romanian Academy, 030304 Bucharest, Romania; 10 Research Institute of the University of Bucharest (ICUB), University of Bucharest, 060023 Bucharest, Romania

**Keywords:** colorectal cancer, microbiota, immune response, intestinal barrier integrity, therapy

## Abstract

Colorectal cancer (CRC) is a multifactorial disease increasingly recognized for its complex interplay with the gut microbiota. The disruption of microbial homeostasis—dysbiosis—has profound implications for intestinal barrier integrity and host immune function. Pathogenic bacterial species such as Fusobacterium nucleatum, Escherichia coli harboring polyketide synthase (pks) island, and enterotoxigenic Bacteroides fragilis are implicated in CRC through mechanisms involving mucosal inflammation, epithelial barrier disruption, and immune evasion. These pathogens promote pro-tumorigenic inflammation, enhance DNA damage, and suppress effective anti-tumor immunity. Conversely, commensal and probiotic bacteria, notably Lactobacillus and Bifidobacterium species, exert protective effects by preserving epithelial barrier function and priming host immune responses. These beneficial microbes can promote the maturation of dendritic cells, stimulate CD8^+^ T cell cytotoxicity, and modulate regulatory T cell populations, thereby enhancing anti-tumor immunity. The dichotomous role of the microbiota underscores its potential as both a biomarker and a therapeutic target in CRC. Recent advances in studies have explored microbiota-modulating strategies—ranging from dietary interventions and prebiotics to fecal microbiota transplantation (FMT) and microbial consortia—as adjuncts to conventional therapies. Moreover, the composition of the gut microbiome has been shown to influence the responses to immunotherapy and chemotherapy, raising the possibility of microbiome-informed precision oncology therapy. This review synthesizes the current findings on the pathogenic and protective roles of bacteria in CRC and evaluates the translational potential of microbiome-based interventions in shaping future therapeutic paradigms.

## 1. Introduction

Colorectal cancer (CRC) is one of the most commonly diagnosed cancers and represents the leading cause of cancer-related mortality. While its pathogenesis involves complex interactions between genetic predisposition and environmental factors, emerging evidence highlights the major contribution of the gut microbiota in modulating colorectal carcinogenesis [1,2]. In the last decade, significant efforts and advancements have been made to comprehend the intricate relationship between the human gut microbiome and CRC, but due to multi-factorial interactions, the precise mechanisms underlying this relationship remain unclear. The intestinal microbiome, comprising trillions of microorganisms, contributes to host homeostasis under normal conditions. The emergence of an imbalance in the microbiota, known as dysbiosis, disrupts intestinal homeostasis and drives chronic inflammation, genotoxicity, and immune dysregulation, thereby promoting tumor development. Dysbiosis is characterized by a decrease in the level of beneficial bacteria, such as *Bifidobacterium* spp. and *Lactobacillus* spp., accompanied by an excessive growth of pathogenic, harmful bacteria, such as *Clostridium difficile* (*CD*), *Fusobacterium nucleatum* (*Fn*), or *Escherichia coli* (*E. coli*) [3,4]. In the context of colorectal cancer (CRC), research has yet to identify a single pathogenic agent. Recent advancements in the fields of microbiology, immunology, and oncology suggest that CRC should be viewed as a condition influenced by the complex interactions between the host and its microbiome rather than being caused by a specific infectious agent [5]. The diverse bacterial species that constitute the microbiota coexist with the host and engage in continuous interactions with immune and epithelial cells, resulting in a bidirectional communication process. The immune response of the host can shape the composition of the microbiota, while the microbiota simultaneously exerts an influence over the host’s immune system. The dysbiosis observed in CRC is not attributable to a solitary microbe but rather arises from an imbalanced ecosystem that compromises the integrity of the intestinal barrier, promotes chronic inflammation, and affects DNA stability [6]. A consortium of bacterial species—including *Fn*, *Enterotoxigenic bacteroides fragilis* (ETBF), *Streptococcus gallolyticus* (*S. gallolyticus*), *Enterococcus faecalis*, toxigenic *E. coli* (phylogroup B2), *Helicobacter pylori* (*H. pylori*), and *Clostridium septicum* (*CS*)—has been implicated in CRC pathogenesis. These microorganisms contribute to tumorigenesis via diverse but often converging mechanisms such as chronic inflammation, genotoxicity, epigenetic reprogramming, immune modulation, and the disruption of epithelial integrity. Rather than acting independently, many of these species may function synergistically, modifying the intestinal microenvironment in ways that favor malignant transformation and progression [7].

Although there is still no unanimously accepted sequence of the appearance of pathogenic bacteria in the dysbiosis that precedes colorectal cancer, some longitudinal studies and animal models suggest a probable three-stage pattern: (1) the pre-neoplastic phase associated with the appearance of dysbiosis due to environmental factors (high-fat diet, antibiotics, obesity) that reduce the abundance of beneficial bacteria (such as *Roseburia* spp.) allowing the development of pathogens [8]; (2) early colonization with “inflammophiles”, pathogenic species that settle in an already permeable epithelium generating oxidative stress and DNA damage, accelerating the transformation of adenoma into carcinoma [9]; (3) the overlap of “tumor promoters” at the time of tumor formation, when the microenvironment becomes hypoxic and rich in necroinflammatory nutrients, proliferating bacteria that can amplify local inflammation and tumor proliferation (for example, *Fn* and *ETBF* and, in advanced stages, *S. gallolyticus* and *CS* (opportunistic species that thrive in necrotic and hypoxic tumor tissue)) [10]. The phases are not strictly sequential and may vary from one individual to another; rather, they represent an ecological dynamic in which niches created by one microbial group are exploited by the next [11,12]. A complex network of interactions facilitates the relationship between the immune system and the microbiota, which is essential for maintaining an equilibrium between immune tolerance and immunogenicity [13]. The immune system develops two distinct types of immune responses, innate and adaptive, which act synergistically against pathogens. Adaptive immune responses can be mediated by antibodies (humoral immune response) or by cells (cellular immune response) [14], with both of them being modulated by the microbiota in CRC [15]. Certain microbial species can either promote immune evasion and tumor progression or enhance anti-tumor immunity, thereby affecting the disease outcome and response to therapy [16,17,18].

Dysbiosis leads to changes in microbial metabolites, resulting in a decrease in beneficial substances like short-chain fatty acids (SCFAs), particularly butyrate. At the same time, dysbiosis increases the production of harmful metabolites, such as lipopolysaccharides (LPS) [19]. Furthermore, dysbiosis contributes to heightened intestinal permeability, which facilitates the entry of microbial products, such as LPS, into the bloodstream. This process can induce chronic inflammation, a factor that plays a significant role in immune dysregulation [20].

Immunological mechanisms are essential and ubiquitous in CRC development and progression, but they work in synergy with genetic, epigenetic, and metabolic mechanisms. Depending on the bacterium and the host context, one of these mechanisms may become dominant. Understanding these complex and context-dependent interactions is essential for developing personalized therapeutic strategies, such as microbiota manipulation by probiotics, targeted bacteriotherapy, or dietary interventions that restore immune homeostasis and inhibit microbially activated oncogenic pathways [21].

This review seeks to elucidate the impact of the gut microbiota on tumor development and progression from an immunological point of view. The initial section examines how gut microbiota influences immune responses that may either promote or inhibit the progression of CRC. We present the strategies employed by pathogenic bacteria that induce chronic inflammation and immune suppression, as well as the mechanisms through which beneficial bacteria can enhance anti-tumor immunity. Additionally, we analyze microbiota-derived metabolites and their role in immune modulation within the context of CRC, aiming to identify how these metabolites affect the efficacy of CRC therapies.

## 2. The Impact of Bacterial Pathogens on Immune Response and Intestinal Barrier Integrity in CRC

Certain gut bacteria serve as more than passive observers in the context of CRC; they function as active participants in the disease’s progression by altering both host immunity and the architecture of epithelial tissue in ways that facilitate tumor development [22,23]. This section summarizes the current evidence on two interconnected fronts: (1) key strategies used by bacterial pathogens to impact immune modulation and evasion in CRC; (2) the mechanisms through which pathogenic bacteria compromise the integrity of the intestinal barrier in this disease. Together, these pathogenic maneuvers create a permissive niche in which neoplastic clones can emerge, expand, and metastasize.

### 2.1. Key Strategies by Which Bacterial Pathogens Modulate and Evade Immunity in CRC

**Figure 1:**
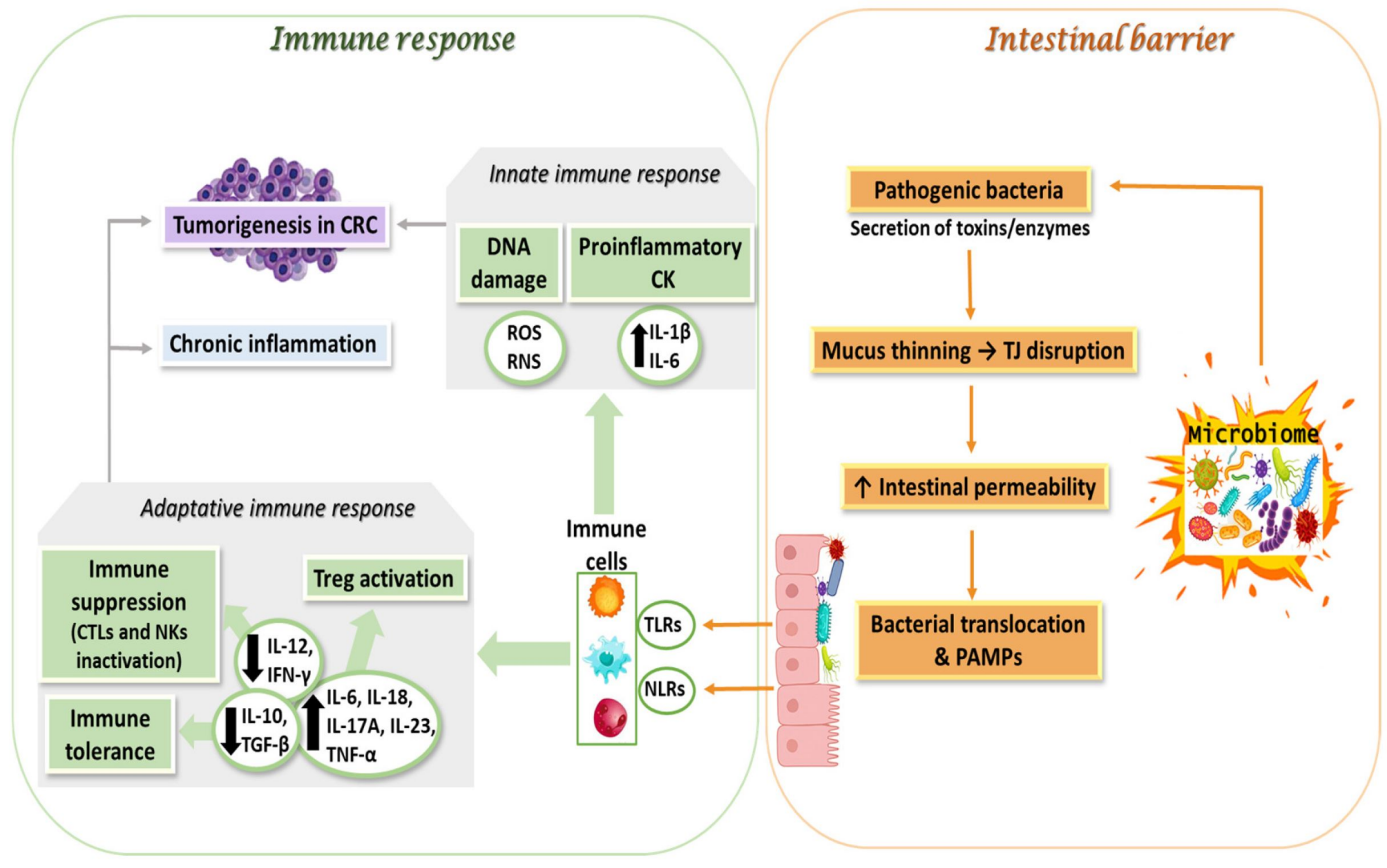
Pathogen bacterial species from microbiome impact on immune response and intestinal barrier integrity in CRC (TLRs—Toll-like Receptors, NLRs—Nucleotide-binding and Oligomerization Domain (NOD)-like Receptors, TJ—Tight Junction, PAMPs—Pathogen-Associated Molecular Patterns (bacteria, viruses, fungi), IL—Interleukin, IFNg—Interferon Gamma, TGFb—Transforming Growth Factor Beta, ROS—Reactive Oxygen Species, RNS—Reactive Nitrogen Species, Treg—Regulatory T cells, CTLs—Cytotoxic T Lymphocytes, NKs—Natural Killer Cells).

Colorectal carcinogenesis unfolds within a microenvironment in which host immunity is continually shaped by the gut microbiota. Pathogenic bacteria exploit this context through a repertoire of immune-oriented strategies that both activate and evade host defenses, ultimately tipping the balance toward tumor promotion via: (1) the activation of innate immune responses, (2) the suppression and evasion of adaptive immunity, (3) crosstalk among pathogenic bacteria, reactive oxygen species (ROS) production, and the immune response (Figure 1).

#### 2.1.1. Activation of Innate Immune Responses by Pathogenic Bacteria

The activation of the innate immune response represents the initial line of defense the body employs against pathogenic incursions. This process is critical for the recognition and response to harmful microorganisms [24]. Pathogenic bacteria can activate the innate immune system by interacting with pattern recognition receptors (PRRs) on immune and epithelial cells, triggering pro-inflammatory signaling pathways [25,26]. Some pathogenic bacteria release molecules such as lipopolysaccharides (LPS), flagellin, or peptidoglycans that bind to receptors on epithelial cells and immune cells (e.g., toll-like receptor 4—TLR4, toll-like receptor 5—TLR5) and trigger the activation of the nuclear factor-κB (NF-κB) and mitogen-activated protein kinase signaling pathways (MAPK) that lead to the increased synthesis and release of pro-inflammatory cytokines, such as interleukine-6 (IL-6), interleukine-1beta (IL-1β), and tumor necrosis factor alpha (TNF-α) [27]. Thus, the generated inflammatory process is sustained and contributes to DNA damage, epithelial barrier disruption, and tumor progression. The activation of nucleotide-binding and oligomerization domain (NOD)-like receptors (NLRs) in epithelial cells by pathogens such as ETBF or certain strains of *E. coli* leads to the formation of the inflammasome (e.g., the NLRP3 inflammasome) and the release of IL-1β and IL-18, which support chronic inflammation and tumorigenesis [28,29]. In addition, as a consequence of the increased synthesis of pro-inflammatory cytokines, there is a massive recruitment of myeloid cells, including macrophages and neutrophils, which produce reactive oxygen species (ROS) and reactive nitrogen species (RNS) in large quantities, leading to DNA damage in epithelial cells [30]. Consequently, chronic inflammation promotes angiogenesis by increasing the production of pro-angiogenic factors, such as vascular endothelial growth factor (VEGF), supporting tumor growth (Figure 1).

#### 2.1.2. Immune Suppression and Evasion

The suppression and evasion of adaptive immunity encompass the mechanisms employed by pathogens to evade or diminish the body’s specific immune response. This phenomenon typically occurs subsequent to the activation of the adaptive immune system. T cells, including CD4^+^ helper cells and CD8^+^ cytotoxic cells, as well as B cells, play a role in inhibiting the presentation of major histocompatibility complex (MHC) molecules. They also contribute to the suppression of the immune response through the action of regulatory T cells (Tregs). As a result of these interactions, pathogens can evade detection, creating conditions that allow for chronic infections [31]. Pathogenic bacteria like *Fn* can impair the function of anti-tumor immune cells. Fn interacts with immune cells through the fusobacterium adhesin A (FadA) adhesin, which binds to E-cadherin on tumor cells, activating Wnt/β-catenin signaling and promoting cell proliferation and invasion. Also, *Fn* expresses the fibroblast activation protein-2 (Fap2), which binds to the immune inhibitory receptor known as T cell immunoreceptor with Ig and ITIM domains (TIGIT) on T cells and natural killers (NK) cells, inhibiting their functions. The result of this process is reduced tumor cell killing and allowing cancer cells to evade immune surveillance. Pathogens may recruit or induce populations of immune cells that facilitate tumor growth and suppress anti-tumor immunity [32]. For example, Fn recruits myeloid-derived suppressor cells (MDSCs) and regulatory T cells (Tregs), both of which suppress cytotoxic T lymphocyte (CTL) functions, which are crucial for attacking tumor cells (Figure 1). Additionally, Fn secretes various factors that impair antigen-presenting cells (APCs), like dendritic cells, preventing an effective anti-tumor immune response (Table 1).

Pathogenic bacteria produce metabolites that interfere with immune functions. Bacterial toxins like colibactin from *E. coli* not only damage DNA but also lead to chronic inflammation which promotes immunosuppressive cell recruitment, making the immune system less effective at targeting tumor cells. These bacterial toxins contribute to promoting immune evasion in CRC (Table 1). Metabolites produced by certain bacteria, like secondary bile acids (e.g., deoxycholic acid), promote oxidative stress and DNA damage [40]. In addition, hydrogen sulfide (H2S) generated by sulfur-reducing bacteria damages epithelial DNA and impairs mitochondrial function. These toxins and metabolites create a pro-carcinogenic environment and enhance tumor evolution [41]. Pathogenic bacteria can suppress anti-tumor immune responses, allowing tumors to grow unchecked and spread to distant sites.

Pathogenic bacteria alter the balance between pro-inflammatory and anti-inflammatory cytokines in the tumor microenvironment. Many bacteria trigger the release of IL-10 and TGF-β, which suppress effector T cells and promote immune tolerance. At the same time, they inhibit interferon-gamma (IFN-γ) and TNF-α, which are crucial for the activation of macrophages, dendritic cells, and T cells that attack tumors. Reduced levels of IFN-γ and IL-12 impair the activation of CTLs and NK cells. Persistent inflammation can exhaust effector T cells, promote Treg activity, and create a tumor-promoting cytokine milieu [42,43]. This shift in cytokine balance makes the tumor microenvironment immunosuppressive, allowing cancer cells to evade immune attack.

#### 2.1.3. Interactions Between the Pathogenic Bacteria, ROS Production, and the Immune Response in Colorectal Cancer

Elucidating how the gut microbiota drives ROS generation reveals that these microbes shape colorectal carcinogenesis by stimulating pro-tumor inflammation, reprogramming immune cells toward a suppressive phenotype, and facilitating tumor immune evasion [44]. Some of the pathogenic bacteria that significantly influence the production of ROS in CRC initiation and progression, and several interconnected mechanisms are presented in Table 2. These bacteria contribute either directly through toxins or genotoxins or indirectly modulating host cells to produce them by inducing inflammation that promotes ROS release by immune cells [45].

*Bacteroides fragilis* (particularly *enterotoxigenic B. fragilis*) releases BFT, which activates nicotine adenine dinucleotide phosphate oxidase (NOX) in colon epithelial cells, leading to ROS production. *E. coli* strains with the pks+ island produce colibactin, a genotoxin that induces DNA double-strand breaks, partly through ROS-mediated mechanisms. These ROS can cause mutagenesis (DNA damage), lipid peroxidation, and protein oxidation that contribute to genomic instability, a hallmark of cancer initiation. In early CRC, microbial ROS activation may break the tolerance of the gut immune system leading to the over-activation of innate immunity [51].

Immune system interactions with microbial imbalance lead to chronic inflammation, and pathogenic bacteria facilitate the infiltration of inflammatory cells like neutrophils and macrophages in the colon mucosa and release high levels of ROS. Chronic exposure to this oxidative environment promotes the oncogenic mutations and activation of pro-cancer signaling pathways (e.g., NF-κB, or Signal Transducer and Activator of Transcription 3-STAT3) [52]. In late-stage CRC, some pathogen bacteria increase oxidative stress which suppresses anti-tumor immunity, for example, *Fn* inhibits NK and T cell activity. However, ROS can suppress APCs and impair dendritic cell (DC) function [53].

In addition, ROS alters immune cell function and polarization. Low ROS levels activate immune responses, including antigen presentation and pro-inflammatory cytokine release. High levels of ROS induce T cell exhaustion, impair CTLs, promote differentiation of Tregs and Th17 cells (linked to tumor growth) or lead to M2-like macrophage polarization (pro-tumor), rather than M1 (anti-tumor) [54,55]. In consequence, ROS reprogram the immune landscape to support immune evasion and tumor tolerance (Table 2).

Some pathogenic bacteria act to induce antioxidant pathway suppression, downregulating host antioxidant enzymes like glutathione peroxidase (GPx) and superoxide dismutase (SOD). This increases intracellular ROS, pushing epithelial cells toward transformation. *Fusobacterium nucleatum*, *E. coli*, and *B. fragilis* together create a biofilm and facilitate a hypoxic, inflammatory microenvironment that promote sustained ROS production at the mucosal surface. This further encourages epithelial-to-mesenchymal transition (EMT), tumor growth, and invasion. ROS can contribute to more microbial translocation and dysbiosis but when ROS is locally higher concentrated ROS feedback appears to alter the microbiome and form a vicious cycle responsible for CRC progression [56].

Understanding these relationships between the microbiome, ROS production, and the immune response can help pave the way for innovative therapeutic strategies and enhance our approach to prevention and treatment.

### 2.2. Mechanisms by Which Pathogenic Bacteria Disrupt the Integrity of the Intestinal Barrier in CRC

The intestinal epithelial barrier is a crucial defense system comprising various types of epithelial cells. This barrier is supported by a thin layer of connective tissue known as the lamina propria, which facilitates communication between the microbiome and immune cells. Additionally, the intestinal epithelial system hosts immune cells, including dendritic cells, T cells, B cells, and macrophages. Together with the epithelial cells, these immune cells contribute to the maintenance of intestinal homeostasis [57].

**Figure 2:**
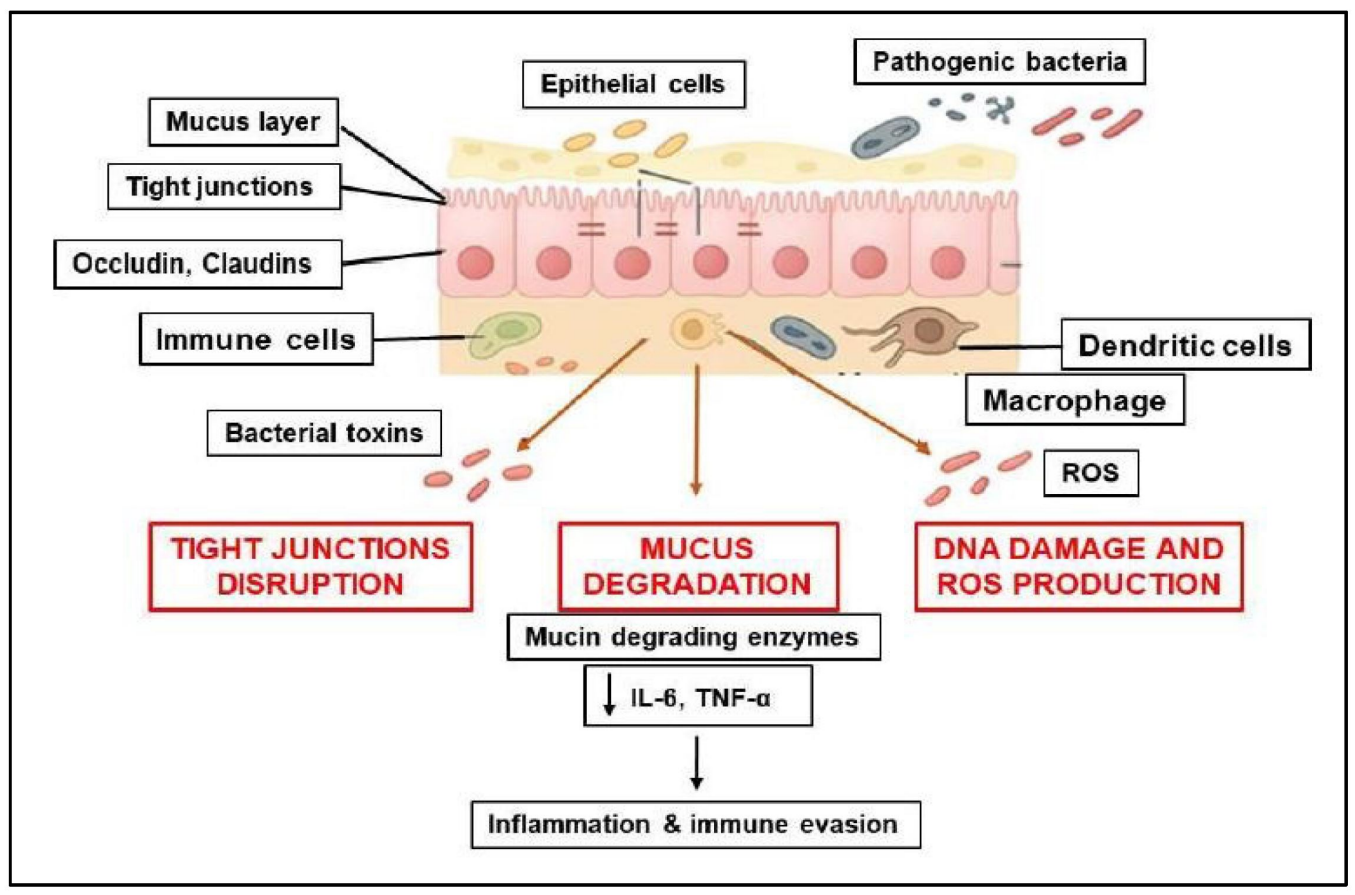
Mechanisms by which pathogenic bacteria compromise the integrity of the intestinal barrier in colorectal cancer CRC.

Pathogenic bacteria can act as harmful agents that compromise the integrity of the intestinal barrier in CRC through several mechanisms, including: (1) disrupting tight junctions, (2) degrading the mucus layer, (3) inducing inflammation, (4) secreting toxins and causing genotoxic effects. As a result, these actions lead to increased permeability, chronic inflammation, and changes in the intestinal microenvironment that promote tumor development [58] (Figure 2).

#### 2.2.1. Disruption of Tight Junctions and Epithelial Integrity

Bacterial pathogens can contribute to tumorigenesis by disrupting tight junctions, increasing intestinal permeability, and triggering inflammation. BFT produced by *Bacteroides fragilis* cleaves E-cadherin, disrupts tight junction proteins (such as ZO-1, occludin, and claudins), and increases epithelial permeability, facilitating tumor development [59,60].

*Fusobacterium nucleatum* can activate inflammatory pathways, including NF-κB and β-catenin, which lead to the downregulation of tight junction proteins and a weakening of barrier integrity [61]. Furthermore, *F. nucleatum* enhances bacterial invasion and colorectal cancer progression through the adhesin FadA, which binds to E-cadherin and disrupts tight junctions. Additionally, *Escherichia coli* (pks+ strains) secrete colibactin, which can modify epithelial adhesion molecules and impact both DNA integrity and epithelial barriers [62].

#### 2.2.2. Degradation of the Mucus Layer

The mucus in the intestinal epithelium contains a highly glycosylated polymeric protein called mucin, which reduces the exposure of epithelial cells to bacteria and viruses from the human microbiome. Recent studies have provided compelling evidence for the significant role of the intestinal microbiota at both the metabolic and immunological levels [63]. *Akkermansia muciniphila* contributes under dysbiotic conditions to the degradation of mucins, thinning the protective mucus layer and thus allowing pathogens to reach the epithelial surface. Some species of *Bacteroides* spp. can act to degrade mucins, exposing epithelial cells to bacterial toxins and inflammatory signals. *S. gallolyticus* also produces virulence factors that promote bacterial adhesion and disrupt epithelial integrity [64].

#### 2.2.3. Effects of the Chronic Inflammation Process Induced by Bacterial Pathogens on Epithelial Integrity

The intestinal barrier, composed of the intestinal mucosa, epithelial cells, and a protective mucus layer, plays a critical role in regulating the passage of substances between the intestine and the bloodstream while preventing the entry of pathogens and endotoxins. The degradation of this barrier by pathogenic bacteria can significantly influence the immune response in CRC. Both systemic and local immune activation, driven by various molecular and cellular mechanisms, are associated with the disruption of the intestinal barrier [65]. In CRC, it has been demonstrated that specific pathogens, including *Fn*, *B. fragilis*, and *E. coli*, facilitate the translocation of bacteria across the mucosa, thereby initiating a local inflammatory response. This process compromises the integrity of the intestinal barrier and is linked to intestinal dysbiosis, which may contribute to tumor progression [66].

When pathogenic bacteria, such as *CD*, *E. coli*, and *B. fragilis*, compromise the intestinal barrier, they can create epithelial lesions that enable the penetration of endotoxins and bacteria. This process can initiate a systemic inflammatory response, highlighting the importance of maintaining intestinal health. Key to this response are pattern recognition receptors (PRRs), including TLR4 (which recognizes LPS), NOD1/NOD2 (which detect peptidoglycan), STING, and cGAS pathway (which respond to bacterial DNA). The activation of these receptors triggers significant intracellular pathways, such as NF-κB and IRF3, ultimately leading to the production of inflammatory cytokines. For example, the bacterium *Fn* activates the NF-κB pathway through TLR4, resulting in the increased secretion of IL-6 and TNF-α, which can negatively impact epithelial integrity. Moreover, bacterial components like LPS, flagellin, and BFT engage TLR4/NF-κB signaling, further contributing to the release of pro-inflammatory cytokines (including IL-6, TNF-α, and IL-1β) that can disrupt the intestinal barrier [67,68]. In response to these challenges, macrophages and dendritic cells act as APCs. The cells capture bacteria or their components, undergo a process of activation, and subsequently migrate to the lymph nodes, where they present antigens to T lymphocytes. This process triggers the activation of CD4^+^ (helper) and CD8^+^ (cytotoxic) T lymphocytes, facilitating a robust adaptive immune response. By comprehending these mechanisms, we can develop strategies that promote intestinal health and enhance immune function [69,70,71] (Figure 1).

Also, dysbiosis accompanied by inflammation generates a polarization of T cells, translated by favoring Th17 cells involved in tumor angiogenesis and Th1 cells responsible for the secretion of pro-inflammatory cytokines associated with the reduction in the Treg population. This immune imbalance process favors the establishment of chronic inflammation and the suppression of immunological tolerance [72,73]. For example, ETBF and *Fn* activate the IL-17/Th17 pathway, which is responsible for the increase in IL-17 secretion. This cytokine is responsible for promoting chronic inflammation and intestinal barrier damage, thus creating a microenvironment favorable to tumor development [74]. *E. coli* pks+, capable of secreting colibactin, intervenes in the activation of the Wnt/β-catenin pathway and TGF-β that affects EMT and induces intestinal barrier dysfunction, thus promoting CRC progression [75,76]. At the same time, systemic inflammation contributes to the repeated stimulation of the immune system, which can result in T cell exhaustion and reduced effective anti-tumor response, favoring tumor progression [77].

#### 2.2.4. The Correlation Between Microbiome, ROS Production, and Barrier Disruption in CRC

When the intestinal flora’s homeostasis is disrupted, many harmful bacteria can induce the oxidative stress-related pathway in the host cell, leading to complex interactions, such as the abnormal differentiation and apoptosis of intestinal cells, the disruption of the epithelial barrier, and inflammatory response (Table 2). Pathogenic bacteria such as *Bacteroides fragilis* ETBF, *E. coli* (pks+), and *Fn* invade the intestine and release toxins (e.g., BFT, colibactin), triggering inflammation. This process causes increased ROS production by host epithelial cells (via the activation of NOX enzymes) or by activated immune cells such as macrophages and neutrophils. Increased ROS production causes oxidative damage to DNA, proteins, and lipids and promotes genomic instability. In addition, high levels of ROS induce epithelial stress and apoptosis, contributing to the weakening of the intestinal barrier and the amplification of the process [78]. The disruption of the intestinal barrier is accompanied by the loss of tight junction proteins (e.g., ZO-1, E-cadherin), a thinning of the mucus layer, and epithelial cell death. As a consequence of these processes, microbial translocation into the submucosa occurs, and immune cells are exposed to bacterial antigens, which results in an amplification of the immune response followed by the increased secretion of TNF-α and IL-6, but also an increase in ROS production, which will generate more tissue damage. Consequently, a weakened barrier allows more pathogenic bacteria to infiltrate and colonize; thus, the cycle repeats and intensifies. These processes facilitate the accumulation of DNA mutations, result in immune suppression, and contribute to the progression of cancer [79].

**Figure 3:**
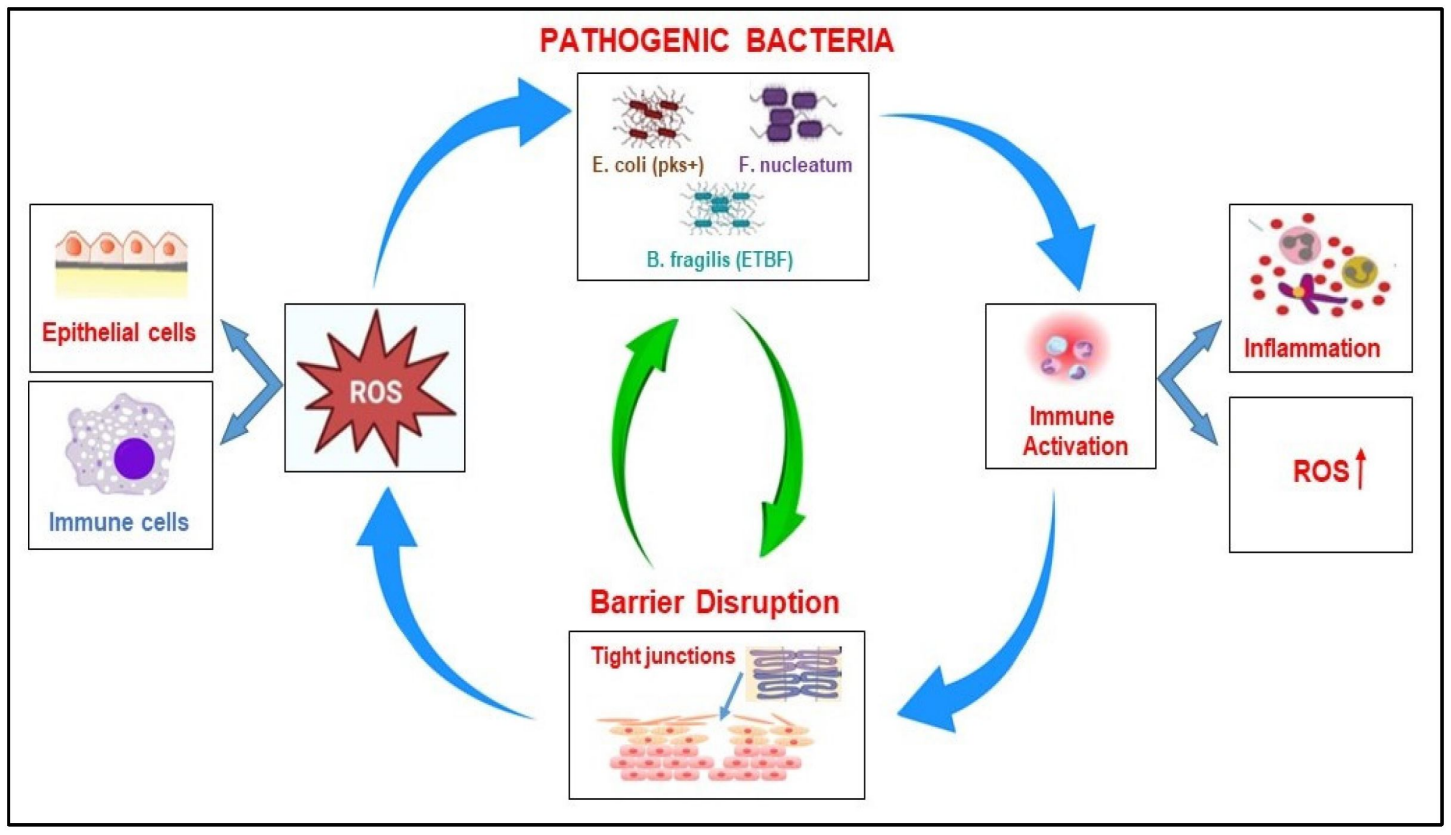
The correlation between pathogenic bacteria, ROS production, immune response, and barrier disruption in CRC; (ROS—reactive oxygen species).

The detrimental cycle involving pathogenic bacteria, the production of ROS, and the disruption of the intestinal barrier in CRC represents the central loop to tumor initiation, chronic inflammation, and progression (Figure 3).

Clearly, these mechanisms collectively weaken the intestinal barrier, promote chronic inflammation, and create a microenvironment favorable for tumor development. Understanding and combating these mechanisms is a crucial task for our audience in the fields of microbiology, oncology, and gastroenterology.

## 3. Beneficial Bacteria and Their Role in Activating Anti-Tumor Immune Defenses in CRC

While pathogenic microbes can sustain colorectal tumorigenesis, a distinct cohort of commensal and probiotic bacteria counterbalances this threat by strengthening host immunity. These beneficial species, such as *Faecalibacterium prausnitzii*, *Bifidobacterium* spp., *Lactobacillus* spp., and *Akkermansia muciniphila*, can modulate immune responses through various mechanisms that activate anti-inflammatory pathways, increase the efficiency of anti-tumor immunity, and support the immune system’s balance. These actions help maintain the integrity of the intestinal barrier and modulate innate and adaptive immunity, ensuring the counteraction of the effects of pathogenic bacteria on tumor promotion [80,81]. Therefore, knowledge of these mechanisms may lead to the development of therapies that directly address the microbiome through new promising approaches to improve CRC treatment outcomes. The most important beneficial bacterial species that play a role in modulating the immune response in CRC are presented in Table 3.

### 3.1. Key Strategies Used by Beneficial Bacteria to Enhance the Anti-Tumor Immune Response in CRC

**Figure 4:**
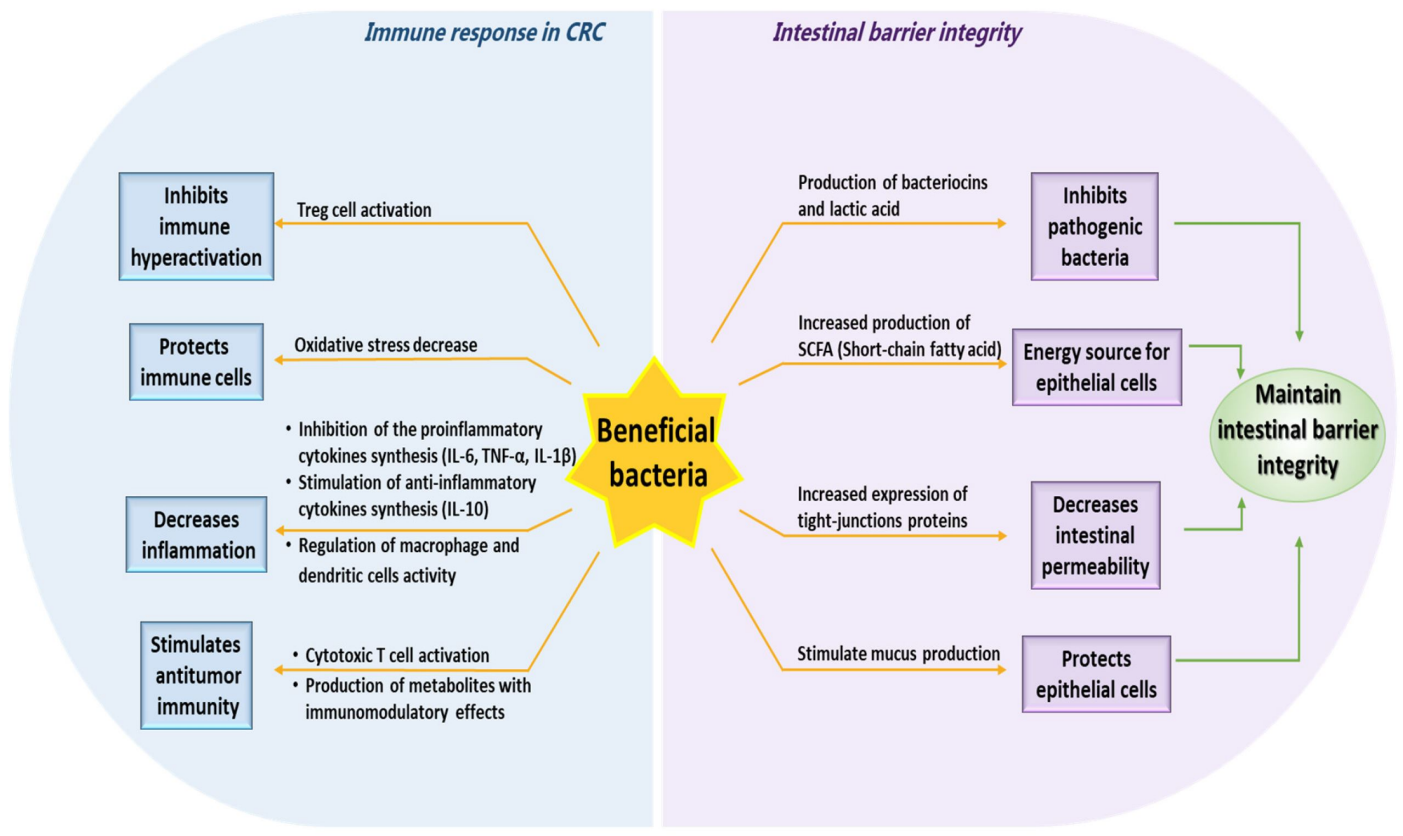
The impact of beneficial bacterial species on immune response and intestinal barrier integrity in CRC.

Beneficial gut microbes counteract colorectal carcinogenesis through several converging immunological tactics: (1) the modulation of the immune response by SCFAs; (2) the induction of anti-tumor immunity and the regulation of innate immunity; (3) the modulation of dendritic cell function; (4) strengthening the intestinal barrier (Figure 4).

#### 3.1.1. Modulation of the Immune Response by SCFAs

Beneficial bacteria, such as *Bifidobacterium* and *Lactobacillus*, produce SCFAs like butyrate, propionate, and acetate through the fermentation of dietary fiber. These SCFAs and other bacterial metabolites suppress NF-κB activation and reduce inflammation by suppressing pro-inflammatory cytokines, such as IL-6 and IL-1β, while simultaneously promoting the production of anti-inflammatory cytokines such as IL-10 [96]. Moreover, SCFAs have a significant impact on the differentiation, recruitment, and activation of immune cells, including neutrophils, DCs, macrophages, and T lymphocytes [97]. Furthermore, SCFAs facilitate the differentiation of regulatory Tregs, which are essential for the modulation of chronic inflammation. They also play a crucial role in the differentiation of regulatory Tregs, which are important for controlling chronic inflammation. Additionally, SCFAs are involved in epigenetic regulation by inhibiting histone deacetylases (HDACs) [98], which enhances the expression of anticancer genes and helps to slow the progression of CRC [99,100]. Beneficial bacteria play a vital role in repairing and regenerating damaged epithelial cells. SCFAs enhance the expression of antimicrobial peptides on the external surface of epithelial cells and modulate their production of immune mediators, including IL-18, which is critical for the maintenance and restoration of epithelial integrity [101]. Notably, butyrate, a type of SCFA, activates the Wnt signaling pathways that promote the proliferation and differentiation of epithelial cells. This process is essential for the continuous renewal of the epithelial layer, which helps maintain barrier integrity in CRC [102].

#### 3.1.2. Induction of Anti-Tumor Immunity and Regulation of Innate Immunity

Some bacteria directly activate anti-tumor immune responses. Beneficial bacteria can promote immune tolerance by stimulating regulatory Tregs or encouraging a balanced immune response between Th1 and Th2 cells, which is important for anti-tumor effects [103]. Furthermore, CTLs can directly activate anti-tumor immune responses. This activation can be enhanced by improving antigen presentation, such as with Bifidobacterium, or by increasing the cytotoxic activity of NK cells, both of which help destroy tumor cells [104]. Additionally, these beneficial bacteria can influence the regulation of innate immunity, helping to maintain immune homeostasis. They do this by encouraging macrophage polarization towards the anti-inflammatory M2 phenotype and by modulating neutrophil activity to prevent excessive tissue damage and inflammation [105,106].

#### 3.1.3. Modulation of Dendritic Cell Function

Immune system cells receive signals from various bacterial substances, which integrate and initiate the activation of appropriate immune response mechanisms. DCs exhibit a broad spectrum of receptors that facilitate the recognition of various bacterial constituents. For instance, specific free fatty acid receptors (FFARs) are capable of detecting bacterial metabolites classified as SCFAs. The activation of these receptors serves as an indication of the bacteria’s viability and demonstrates how their metabolites interact with the immune system to promote anti-inflammatory functions [107]. Furthermore, the diverse array of receptors expressed by DCs enables them to activate multiple signaling cascades concurrently. This ability to integrate several signals simultaneously ensures the generation of an appropriate response to both antigenic and tissue-related stimuli [108]. The modulation of dendritic cell function by beneficial bacteria influences DCs to act as antigen-presenting cells for T cells. Consequently, beneficial bacteria promote the development of tolerogenic DCs, which enhance the induction of Treg cells or stimulate DCs to produce anti-inflammatory cytokines and prevent the over-activation of the immune system [109]. Furthermore, these bacteria can stimulate dendritic cells to produce anti-inflammatory cytokines, such as IL-10, thereby contributing to the balance of immune responses and preventing the excessive activation of the immune system, particularly in the context of CRC [110].

#### 3.1.4. Strengthening the Intestinal Barrier

The intestinal barrier is a crucial barrier for an organism against invasion by foreign pathogens, including mechanical, chemical, immune, and microbial barriers. Beneficial bacteria play a crucial role in strengthening the intestinal barrier in CRC by maintaining intestinal homeostasis and reducing inflammation (Figure 4). *Lactobacillus*, *Bifidobacterium*, and *Akkermansia muciniphila* act beneficially and promote mucin secretion by goblet cells, forming a protective barrier that prevents the entry of pathogens and minimizes the activation of the immune system, thus promoting overall gut health [111].

Several beneficial bacteria (*Lactobacillus rhamnosus GG*, *Lactobacillus plantarum*, Lactobacillus reuteri, Bifidobacterium breve, Bifidobacterium longum, *Bifidobacterium bifidum*, *Akkermansia muciniphila*, and *Faecalibacterium prausnitzii*) play a significant role in improving the integrity of the intestinal epithelial barrier by upregulating the expression of tight junction proteins (occludin, claudins, and zonula occludens) and preventing CRC progression (Figure 4).

Beneficial bacteria are instrumental in safeguarding the epithelial barrier by scavenging ROS and mitigating oxidative stress. For example, species within the Lactobacillus genus are known to synthesize antioxidants and detoxify harmful metabolites, thereby preventing oxidative damage to epithelial cells [112]. Furthermore, specific bacterial metabolites can diminish epithelial inflammation and reinforce the integrity of the barrier [113]. Indole derivatives resulting from tryptophan metabolism are known to activate the aryl hydrocarbon receptor (AhR) in epithelial cells, facilitating both barrier integrity and immune regulation. Moreover, certain intestinal bacteria possess the capacity to degrade toxic compounds, such as bile acids and polyamines, which are implicated in the progression of CRC [114]. Additionally, beneficial bacteria occupy competing niches, effectively outcompeting harmful bacteria for the occupation of adhesion niches on the mucosa by secreting bacteriocins and organic acids that directly inhibit pathobionts, maintaining a local pH unfavorable to pathogenic species, and preferentially consuming essential nutrients, thus preventing pathogenic bacterial colonization that could compromise the intestinal barrier (Figure 4) [115].

Collectively, these mechanisms underscore the significance of beneficial bacteria in fortifying tight junctions, reducing intestinal permeability, and providing protection against the progression of CRC. These mechanisms emphasize the crucial role of beneficial bacteria in enhancing the integrity of tight junctions, which are vital barriers within the intestinal lining. Furthermore, their protective effects play a significant role in reducing the risk of developing CRC, offering an important defense in the battle against this disease [116].

## 4. Role of the Microbiome in Current and Future Therapeutic Strategies for Colorectal Cancer

The composition of the colorectal microbiota plays a significant role in modulating local inflammation, epithelial permeability, and the efficacy of various treatments, including chemotherapy and immunotherapy. This observation supports the notion that true personalization in colorectal cancer (CRC) management necessitates the consideration of a triad comprising: (1) tumor genotype, (2) immune signature, which includes microsatellite instability (MSI), tumor mutational burden (TMB), and CD8^+^ T cell infiltration, (3) the microbial ecosystem. Each component contributes distinct targets and biomarkers, facilitating the development of genuinely adaptive multimodal therapies. Furthermore, the microbiome is emerging as a critical factor in both the pathogenesis of CRC and the responsiveness to treatment. Consequently, contemporary and future therapeutic strategies increasingly incorporate the microbiome as a target or modulator of treatment efficacy and toxicity.

### 4.1. Microbiome Impact on Current Therapies in CRC

Currently, CRC treatment has become increasingly personalized, combining curative surgery with molecularly guided systemic regimens and, more recently, with immunotherapy. Numerous studies demonstrate that the microbiome has a significant impact on the efficacy, toxicity, and outcomes of current therapies in CRC [117], intervening in a complex way in drug metabolism, immune response, and the development of tumor resistance.

#### 4.1.1. Microbiome Modulation of Chemotherapy Efficacy and Toxicity

Chemotherapy remains the standard first-line treatment in CRC, with an essential role in controlling the disease, and preparing the patient for combination treatments or surgery, where possible. For localized stages (local disease), oncological resection remains the foundation, complemented by chemotherapy (FOLFOX/CAPOX) and, for rectal tumors, preoperative radio- or chemoradio-therapy (“short course” (5 × 5 Gy) or long chemoradiotherapy (50 Gy + capecitabine), to reduce tumor volume and preserve sphincter function) [118]. At the advanced or metastatic stages, chemotherapy is often combined with other types of therapies, depending on the stage of the disease and the molecular profile of the tumor.

Microbiome bacteria can modulate the activation or deactivation of chemotherapy drugs before their delivery to tumors, thereby influencing their efficacy and toxicity. Certain microbial species produce enzymes, such as β-glucuronidases, which can convert inactive drug metabolites back into their active and toxic forms, resulting in adverse side effects. A pertinent example is Irinotecan (CPT-11), which is metabolized in the liver to an inactive form (SN-38G) and subsequently reactivated by bacterial β-glucuronidase, leading to inflammatory responses [119]. Furthermore, the microbiome exerts a considerable influence on immune cell recruitment and the inflammatory pathways that are critical to chemotherapy-induced tumor cell death. Empirical studies have demonstrated that the composition of the microbiota can impact immune responses to treatments such as 5-Fluorouracil (5-FU), thereby modulating both the effectiveness and side effects of the treatment oxaliplatin, another chemotherapeutic agent, which induces tumor cell death through the generation of ROS and enhances the anti-tumor immune response by activating mechanisms of innate immunity [120]. Additionally, specific bacteria, including *Alistipes* and *Lactobacillus*, have been identified as enhancers of the immune-mediated anti-tumor effects associated with oxaliplatin. Conversely, other bacteria may promote chemoresistance through mechanisms such as inflammation, the disruption of the mucosal barrier, or immune system suppression. For instance, *Fn* has been implicated in promoting chemoresistance via pathways that inhibit apoptosis and facilitate autophagy [121].

#### 4.1.2. Microbiome Impact on Immunotherapy

In metastatic disease, treatment is based on the genetic profile of the tumor (RAS, BRAF, HER2, or MSI/dMMR) and the extent of the disease [122]. Programmed cell death 1 (PD-1) immunotherapy (pembrolizumab, nivolumab) has become the standard in patients with microsatellite instability-high (MSI-H) or mismatch repair deficiency (dMMR) tumors, conferring durable responses, and current research is exploring bimodal synergies (ICI and anti VEGF/EGFR, radiotherapy, or microbiota manipulation) for refractory microsatellite stable (MSS) tumors. This multimodal and biomarker-targeted approach transforms CRC from a uniformly treated disease to one managed by sequential strategies tailored to each patient. Anti-PD-1 immunotherapy has decisively changed the treatment paradigm for the subgroup of CRC with MSI-H or dMMR [123]. MSI H/dMMR tumors accumulate thousands of “frameshift” mutations that generate abnormal peptides, neoantigens, which are processed by the proteasome, loaded on major histocompatibility complex-1 (MHC I) molecules, and presented on the surface of tumor cells. Due to their uniqueness, they are recognized as non-self by naive T lymphocytes in the mesenteric nodes, inducing intense proliferation and differentiation into effector cells in CTLs. The large number and diversity of these neoantigens leads to the polyclonal clonal expansion of distinct T cells, each with a unique specificity, from which some dominant clones are selected and multiplied that exert a strong immune pressure on the tumor, secreting IFNγ and TNFα and releasing granzymes/perforin that induce the apoptosis of malignant cells [124]. In the absence of PD-1 blockade, the response is rapidly exhausted by tumor evasion mechanisms, and the therapeutic inhibition of PD-1 re-energizes these expanded clones and prolongs their activity. PD-1 inhibition restructures the tumor microenvironment: re-energized CTLs secrete IFN-γ that polarizes macrophages towards an M1 phenotype and stimulates stromal cells to secrete the chemokines CXCL9/10, which additionally attract NK cells to the tumor [125]. Concomitantly, the density of suppressor cells, such as Treg and myeloid-derived suppressor cells (MDSCs), decreases [126], either through apoptosis induced by the pro-inflammatory environment or through unfavorable competitive chemotaxis, and MHC-I/II expression on tumor cells increases, enhancing antigen presentation. The synergy between high neoantigen load, clonal expansion, and PD-1 re-energization supports a robust polyclonal response, explaining the durable remissions observed in MSI-H/dMMR patients. For patients with MSI-H/dMMR tumors, the introduction of PD-1 inhibitors has transformed the prognosis from less-than-one-year survival to five-year survival rates exceeding 55% [127], validating the concept of “durable benefit” and establishing immunotherapy as the backbone of treatment in this molecular subset of CRC.

The efficacy of immunotherapy, particularly in relation to immune checkpoint inhibitors (ICIs), can be significantly impacted by the composition of the gut microbiota. Immunotherapy, particularly ICIs, shows great promise in treating CRC that is characterized by MSI-H or dMMR [128]. Conversely, it exhibits limited effectiveness in microsatellite stable (MSS) CRC, unless influenced by the microbiome. Certain beneficial microbes can potentially enhance the therapeutic response to ICIs by improving antigen presentation and promoting T cell activation. For instance, *Akkermansia muciniphila* has been shown to stimulate DCs, thereby augmenting the effectiveness of PD-1 blockade. Additionally, *Bacteroides fragilis* (non-toxigenic) can induce Th1 immunity, which increases the efficacy of anti-cytotoxic T lymphocyte antigen 4 (anti-CTLA-4) treatment. Furthermore, *Faecalibacterium prausnitzii* plays a crucial role in maintaining mucosal integrity and is also noteworthy for highlighting the potential of microbiome modulation in cancer therapies [81]. Moreover, while the use of antibiotics is often necessary, it is important to be aware that their administration before or during ICI therapy can lead to adverse effects on response rates due to gut dysbiosis [129]. Addressing this issue and understanding the implications on the gut microbiota, such as the impact of *Fn* related to immune evasion and therapy resistance, will be crucial in optimizing treatment strategies and improving patient outcomes [130]. By focusing on these microbial interactions, we can potentially enhance the effectiveness of immunotherapy in colorectal cancer patients.

#### 4.1.3. Microbiome Impact on Targeted Therapy

Targeted therapies constitute an essential aspect of CRC management, particularly in cases of metastasis. This therapy involves the use of molecules that block angiogenesis and are known as anti-VEGF (Vascular Endothelial Growth Factor) inhibitors, such as Bevacizumab (Avastin), Ziv-aflibercept (Zaltrap), and Ramucirumab (Cyramza). Furthermore, in the context of RAS wild-type and BRAF wild-type tumors, anti-EGFR (Epidermal Growth Factor Receptor) inhibitors are employed to obstruct signaling pathways that promote cell growth. These agents are marketed under various trade names, including Cetuximab (Erbitux) and Panitumumab (Vectibix). The microbiota may interact with the tumor microenvironment, influencing the responses to anti-EGFR or anti-angiogenic therapies; thus, in metastatic disease, chemotherapy is supplemented with targeted therapies against EGFR or VEGF, and newly approved combinations—encorafenib + cetuximab for BRAFV600E, adagrasib + cetuximab for KRASG12C, and trastuzumab deruxtecan for HER2 positive—expanding the options in advanced lines [131,132,133].

Emerging research on the therapeutic interplay between the gut microbiota and targeted treatments reveals a fascinating landscape where specific gut bacteria, like *Akkermansia muciniphila* and *Bacteroides fragilis*, play pivotal roles. These microorganisms possess the remarkable ability to invigorate immune responses and mitigate inflammation, which may significantly bolster the effectiveness of anti-EGFR therapies. Furthermore, *Bifidobacterium* emerges as a powerful ally, capable of enhancing the therapeutic impact of anti-VEGF treatments [134].

These studies emphasize the increasing interest in the integration of microbiome modulation into treatment strategies for CRC by synergistically blending traditional therapies with innovative interventions designed to influence the gut microbiota. The aspiration is to significantly elevate treatment effectiveness, surmount resistance barriers, and alleviate the burden of adverse effects, ultimately paving the way for more holistic and patient-centered care.

### 4.2. Therapeutic Modulation of the Microbiome in CRC

Combinatory therapies for CRC that integrate microbiome components are part of a rapidly evolving field aimed at enhancing therapeutic efficacy, overcoming resistance, and reducing toxicity. These strategies involve combining conventional treatments (chemotherapy, immunotherapy, and targeted therapy) with microbiome-modulating interventions, such as probiotics, prebiotics, antibiotics, or fecal microbiota transplantation.

#### 4.2.1. Probiotics, Prebiotics, Synbiotics, and Postbiotics in Therapeutic Approaches in CRC

The introduction of microecological agents such as probiotics, prebiotics, synbiotics, and postbiotics, in CRC management hold the potential to modulate gut microbiota. These agents contribute to both the prevention and treatment of CRC by restoring microbial balance, enhancing immune responses, reducing inflammation, and suppressing tumor-promoting pathways.

*Probiotics*, living microorganisms which are specific non-pathogenic strains of bacteria or yeast, are beneficial to human health when consumed in adequate amounts, helping to support a balanced and healthy gut microbiota. They belong to the genus *Lactobacillus* and *Bifidobacterium* but also include *Bacillus*, *Pediococcus,* or yeasts and are found in fermented food and drinks (yogurt, kefir) or as dietary supplements [135]. Certain probiotic strains from the genera *Lactobacillus* and *Bifidobacterium* (especially *Lactobacillus rhamnosus GG*, *Lactobacillus casei,* and *Bifidobacterium longum*) have demonstrated the ability to reduce the activity of pro-carcinogenic bacterial enzymes such as β-glucuronidase, azoreductase, and nitro-reductase, which are involved in the conversion of food or bile pro-carcinogens into active metabolites. Moreover, these probiotics can sequester mutagens (e.g., heterocyclic amines from processed meat), reducing their bioavailability. *Prebiotics*, indigestible fibers or substances found in specific foods that nourish the beneficial bacteria in the gut, support the growth and activity of good bacteria, helping to maintain a healthy digestive system. Inulin [136], fructo-oligosaccharides (FOS), galacto-oligosaccharides (GOS) [137], and pectin [138], are among the most investigated prebiotics, with the aim of evaluating their potential role in CRC prevention and treatment [139].

A recent study underlines that, in contrast to the conventional methods of vaccination that stimulate systemic immune response, the administration of probiotics as oral vaccines holds several advantages, such as the ability to target both systemic and mucosal immunity, convenient storage and transport without the need for refrigeration or cold chain systems, and inherent acid and bile tolerance, which aids survival in the gut environment. Unlike attenuated vaccines, probiotics eliminate the risk of reversion to virulence. Advanced strategies that employ CRISPR-Cas editing, the insertion of heterologous genes into bacterial chromosomes, and Phage-Mu transposition improve the genetic stability of probiotics. Moreover, the association of probiotics with prebiotics, encapsulation, and enhancing acid tolerance leads to improved host colonization, thereby optimizing probiotic vaccine performance [140].

*Synbiotics* combine probiotics and prebiotics to promote a healthy gut microbiome. The synergy of probiotics with prebiotic fibers enhances the production of SCFA and contributes to the restoration of the intestinal barrier integrity (by increasing the expression of zonulin and occludins), reducing bacterial translocation and systemic inflammation, which are critical factors in the progression of CRC. Thus, their administration can modulate the intestinal metabolic profile in a protective sense [141]. In colorectal oncology, *postbiotics*, structural or metabolic products of viable bacteria that exert host benefits even in the absence of the living microorganism, offer the advantage of controllable bioavailability, eliminating the risk of bacterial translocation in immunocompromised patients. Compared with probiotics, postbiotics have been shown to have a safer and more stable chemical structure. Postbiotics include SCFAs (butyrate, acetate, and propionate), enzymes, cell wall fragments (peptidoglycan), bacterial metabolites, exopolysaccharides, bacteriocins, vitamins, and amino acids [142]. A recent article explores the potential of bacteriocins—specifically Lactacin B, Lactacin F, and Doderlin—produced by lactic acid bacteria (LAB) as safe and effective therapeutic agents for colorectal cancer (CRC). The study utilized in silico analysis to identify strong binding affinities between these compounds and key CRC-associated genes, such as CTNNB1 and LRP5. This indicates their potential to modulate the Wnt signaling pathway and to provide a less toxic therapeutic option compared with current CRC treatments [143].

Used both as prevention and adjuvant therapy in CRC treatment, postbiotics exert their anticancer role through multiple mechanisms including the modulation of the intestinal microbiota, the enhancement of the intestinal mucosal barrier, the regulation of the immune response, and their influence on systemic metabolism [144,145].

The incorporation of beneficial microbes or compounds into supplementation strategies serves to promote their growth. This approach not only aids in reducing the inflammation associated with colorectal cancer (CRC) but also enhances the mucosal barrier function and improves tolerance to chemotherapy.

#### 4.2.2. Fecal Microbiota Transplantation (FMT)

FMT is an innovative medical procedure in which fecal matter from a healthy donor is transferred into the intestine of a person with dysbiosis to restore the balance of the intestinal microbiota [146]. FMT contains the bacteria and viruses naturally present in healthy stools. FMT is currently widely accepted and recommended as an effective therapeutic strategy for the management of recurrent *Clostridioides difficile* infection [147], and its use in experimental studies for other intestinal diseases such as inflammatory bowel disease (IBD), constipation, short bowel syndrome (SBS), and irritable bowel syndrome (IBS) showed promising results. In obesity, type 2 diabetes, and metabolic syndrome, FMT is being investigated for its potential to modulate the metabolism and systemic inflammation by intervening in the composition of the intestinal microbiota [148,149]. A growing number of experimental studies are currently exploring the potential of FMT to inhibit the progression of CRC. Using a chemically induced murine model of CRC with intestinal microbial dysbiosis, a recent study [150] investigated the effects of introducing healthy gut bacteria through FMT. This strategy restored the composition and diversity of the gut microbiota in CRC mice, facilitated the recruitment of intestinal immune cells, improved host immune activity targeting CRC, modulated cytokine expression within the CRC microenvironment, and thereby inhibited tumor growth and prolonged survival.

Immunotherapy can enhance the immune system’s ability to recognize and attack cancer cells, while also potentially disrupting tumor growth and metastasis. Combining FMT with pembrolizumab or nivolumab may offer a synergistic effect, improving disease control. A phase II clinical trial, conducted by the MD Anderson Cancer Centre (NCT04729322), is currently exploring the efficacy of FMT in combination with the reintroduction of anti-PD-1 therapy (pembrolizumab or nivolumab) in patients with advanced metastatic CRC who have failed anti-PD-1 therapy [151].

The interaction between the microbiota and radiotherapy is a complex process. Bidirectional anticancer treatments can alter the microbiome, leading to dysbiosis, and these microbial changes, in turn, can affect both the efficacy of treatments and the severity of treatment-related gastrointestinal toxicities. In this context, FMT may be associated with radiotherapy and could restore the diversity of the intestinal microbiota, consequently improving the response to radiation and reducing side effects [152]. These data support the beneficial effect of transferring a healthy microbiome to a patient, enhancing the efficacy of immunotherapy and reducing dysbiosis induced by chemotherapy, radiotherapy, or antibiotics.

#### 4.2.3. Therapeutic Bacteriophages

Bacteriophages, commonly referred to as phages, are specific viruses that infect bacteria and play a vital role in regulating bacterial populations. These phages can be modified to deliver therapeutic agents. Preliminary research and preclinical studies have explored the use of lytic phages targeting *Fusobacterium nucleatum*, a bacterium linked to enhanced tumor cell proliferation and the promotion of chemoresistance by shielding tumors from immune cell attacks. Furthermore, the increased abundance and prevalence of *Fusobacterium nucleatum* correlate with more advanced tumor stages and a poorer prognosis in patients diagnosed with CRC [153,154]. These phage therapies have shown potential in reducing tumor growth in CRC xenograft mouse models achieved through a decrease in bacterial load, a reduction in inflammation, and an increase in T cell infiltration [155].

Innovations derived from the CRISPR-Cas (clustered regularly interspaced short palindromic repeats) immune system of prokaryotes have facilitated the application of CRISPR-Cas tools for human gene editing. These tools may also be employed to target the pathogenic and commensal bacteria that colonize the human body, thereby providing novel avenues for treating infections and modulating the microbiome [156].

Phages can be engineered to selectively delete virulence genes or to eliminate antibiotic-resistant bacteria associated with CRC by utilizing CRISPR-Cas3 or CRISPR-Cas9 technologies. CRISPR-Cas tools possess the capability to eliminate or modify specific bacteria through DNA and RNA targeting strategies, effectively disarming them by deleting, altering, or silencing particular genes [157,158]. Additionally, the delivery of CRISPR-Cas tools via bacteriophages or conjugation highlights promising therapeutic opportunities for treating infectious diseases and modifying the microbiome, underscoring both the advancements achieved and the challenges that remain in translating these methodologies into clinical practice.

One notable advantage of phage therapy is its high specificity, which ensures the targeted elimination of pathogens while preserving the diversity of the gut microbiome, thus offering a potential strategy to address antibiotic resistance. Therapeutic bacteriophage approaches in CRC are an emerging and promising area of research that targets pro-tumor bacteria in the gut, modulates the tumor microenvironment, and potentially enhances the efficacy of existing cancer therapies [159,160].

### 4.3. Perspectives in CRC Therapy

The perspectives in the treatment of CRC encompass the development of novel pharmaceuticals, advancements in immunotherapy, targeting of the microbiome, and the implementation of precision medicine. A primary focus of research is the translation of these innovations into clinical applications to enhance patient outcomes through personalized medicine, early detection, and combination therapies. Research efforts are focused on the comprehensive genomic profiling of CRC, examining mutations in key genes such as KRAS, NRAS, BRAF, MSI, HER2, and PIK3CA to identify new actionable mutations and signaling pathways. A range of targeted therapies is currently being evaluated in clinical trials for specific patient populations, including BRAF mutant CRC therapies utilizing combinations such as encorafenib and cetuximab, HER2-targeted therapies (trastuzumab and pertuzumab) for HER2-positive CRC, and KRAS G12C inhibitors, such as sotorasib [161,162]. Additionally, investigations into the roles of immune cell exhaustion, the tumor microenvironment, and the microbiota have facilitated the clinical translation of various combination therapies. These include ICIs combined with chemotherapy, anti-VEGF agents, and oncolytic viruses [163]. The development of bispecific antibodies, CAR-T cells, and vaccines that target tumor-associated antigens is also underway. Significant research is focused on understanding the impact of microbial influences on drug metabolism, immune modulation, and the development of resistance. This knowledge has led to the clinical application of FMT to improve responses to immunotherapy, as well as the exploration of phage therapy in clinical trials. Furthermore, it is essential to monitor the synergistic effects of combining existing pharmaceuticals with immunotherapy or targeted agents [164]. Ongoing clinical trials are investigating combinations such as ICIs with MEK inhibitors, chemotherapy, or checkpoint inhibitors in the presence of microbiota modulators [165]. Finally, the analysis of circulating tumor DNA (ctDNA) and circulating tumor cells (CTCs) through liquid biopsies is instrumental in enabling the early detection of minimal residual disease (MRD) and in providing critical guidance for therapeutic interventions.

## 5. Challenge and Future Directions

Colorectal cancer represents a major public health problem, and the intestinal microbiota influences both tumor initiation and progression, as well as therapeutic response, transforming itself from a possible risk factor into a true therapeutic resource. The links between the microbiota and colorectal cancer form an extremely complex bidirectional network: bacteria, bacteriophages, and bacterial metabolites interact with the tumor genome, the epithelial barrier, and host immunity.

Multi-cohort studies have shown that a group of pathobionts and the decrease in butyrate-producing bacteria can discriminate patients with adenomas/CRC from healthy controls. Thus, colorectal microbiota profiling has diagnostic potential for establishing a microbial signature for early detection, but has not yet become a “routine” test. Inter-individual variability (diet, antibiotics, or comorbidities), lack of standards for collection/storage, and sequencing costs prevent, for now, large-scale clinical validation. Circulating microbial DNA (cmDNA) from liquid biopsy contains fragments from tumor-associated bacteria; their profiling, correlated with ctDNA mutations, may suggest the colorectal tumor origin from the early stages. Oncologists can use fecal or plasma signatures for risk stratification and for the selection of patients eligible for immunotherapy/synbiotics, making it necessary to integrate microbial biomarkers into oncological decision making. Fecal microbiota profiling is a promising biomarker for screening and risk stratification, but for now, remains complementary to established tests.

The adjuvant role of probiotics/synbiotics/postbiotics in CRC, especially perioperatively and during chemotherapy, through detoxification, barrier strengthening, and immune modulation, is proven. The challenge is the standardization of strains and doses, and the integration of response biomarkers as essential steps towards large-scale clinical implementation. Studies have shown that FMT and bacteriophages can selectively remodel the colonic ecosystem in CRC, but critical barriers remain: FMT risks pathogen transmission and provides variable results between donors, and phage therapy faces pharmacokinetic limitations.

The future depends on: (i) obtaining standardized microbial consortia grown in good manufacturing practice standards, (ii) personalized phage cocktails based on tumor/fecal sequencing, (iii) adaptive clinical trials integrated with immunotherapy. If these obstacles are overcome through regulations and bioengineering, FMT and bacteriophages may become safe and precise adjuvants in the multimodal therapy of colorectal cancer.

Exploring innovative approaches has the potential to facilitate the establishment of comprehensive databases concerning microbiome–drug interactions. By employing machine learning and large language model platforms, it is possible to integrate diverse data types—including genomics, transcriptomics, pathology images, and microbiome data—to determine which drugs a tumor or other diseased tissue may be sensitive to or resistant against. Furthermore, this methodology can assist in predicting prognosis, recurrence risk, and the likelihood of side effects.

## 6. Conclusions

This review provides a comprehensive overview that supports the essential role of the composition and activity of the gut microbiota in colorectal carcinogenesis. Both pathogenic and beneficial microbes influence host immune pathways, which are critical for tumor suppression or promotion. While some pathogens inhibit antigen presentation and cytotoxic T cell function, beneficial bacteria enhance immune surveillance and support immune cell-mediated anti-tumor activity. These processes are intricate and are mediated by microbial metabolites, surface molecules, and signaling pathways that facilitate communication between the host and microbes.

Moreover, we highlighted the significant potential of microbiota as a therapeutic target for CRC. Strategies such as probiotics, prebiotics, dietary modifications, and FMT are currently being investigated to restore microbial balance and improve the efficacy of standard treatments, including immunotherapy and chemotherapy. Integrating microbiome-targeted interventions with conventional and emerging therapies may lead to more effective, durable, and personalized treatment outcomes for CRC. However, it is essential to emphasize that further studies and well-controlled clinical trials are necessary to fully understand the causal relationships between specific microbes and the progression or regression of CRC.

## Figures and Tables

**Table 1:** Pathogenic bacteria impact the immune response and barrier integrity in CRC.

Pathogenic Bacteria	Immune Response	Intestinal Barrier Integrity
*Fusobacterium nucleatum* [33]	- Inhibition of cytotoxic T cell and NK cell activity. - Activates tumorigenic pathways (e.g., Wnt/β-catenin). - Promotes inflammation, adhesion, immune evasion, and β-catenin activation. - Recruit immune cells like myeloid-derived suppressor cells (MDSCs) that promote a tumor-permissive microenvironment.	- Invade colonic epithelial cells, disrupting the epithelial barrier and enhancing tumor cell proliferation. - Downregulating tight junction proteins and weakening barrier integrity.
*Enterotoxigenic Bacteroides fragilis* (*ETBF*) [6]	- Promote Treg activity. - Activate NF-κB signaling. - Induce secretion of pro-inflammatory cytokines like IL-6, IL-17, and TNF-α.	- Produces *Bacillus fragilis toxin* (BFT), which degrades tight junction proteins (e.g., E-cadherin) and increases barrier permeability.
*Salmonella enterica* [34,35]	- Uses its Type III secretion system to inject effectors that trigger inflammation. - Triggers pro-inflammatory responses.	- Induce epithelial barrier damage through its Type III secretion system.
*Akkermansia muciniphila* [36]		- Under dysbiotic conditions, degrades mucins, thinning the protective mucus layer and allowing pathogens to reach the epithelial surface.
*Escherichia coli harboring* the polyketide synthase (pks) [37]	- Produce toxins like colibactin and affect immune cell recruitment and activation, promoting immune evasion. - Induces oxidative stress.	- Alters epithelial adhesion molecules. - Disrupts epithelial barrier integrity, leading to increased permeability and chronic inflammation.
*Helicobacter pylori* [38]	- Activate pro-inflammatory responses.	- Generate virulence factors (*CagA* and *VacA*) that can disrupt epithelial tight junctions, enhancing permeability.
*Streptococcus bovis* (now called *Streptococcus gallolyticus*) [39]	- Induce inflammatory and proliferative pathways.	- Increase intestinal permeability.

**Table 2:** Pathogenic bacteria-induced ROS and their impact on immune response in CRC.

Pathogenic Bacteria	Mechanism of ROS Induction	Effect of ROS on Immune Response in CRC	Resulting Impact on CRC Progression
*Fusobacterium nucleatum* [46]	Activates NOX enzymes and mitochondrial ROS	Suppresses T cell activation; promotes M2 macrophages	Promotes immune evasion and tumor growth
*Escherichia coli* (pks+ strains) [47]	Produces colibactin → DNA damage and mitochondrial ROS	Induces inflammatory cytokines (IL-6, TNF-α); activates NF-κB	Enhances tumor-promoting inflammation
*Enterotoxigenic Bacteroides fragilis* (ETBF) [48]	Induces ROS via epithelial NADPH oxidase	Skews CD4^+^ T cells toward Th17 phenotype (IL-17 production)	Promotes chronic inflammation and CRC
*Salmonella enterica* [49]	Activates ROS through host immune signaling (e.g., TLRs)	Induces pro-inflammatory cytokines, disrupts gut barrier	Facilitates tumor-promoting microenvironment
*Helicobacter pylori* (colon involvement rare but possible) [50]	Cytotoxin-associated gene A (*CagA*) protein and vacuolating cytotoxin A (*VacA*) induce oxidative stress	Upregulates inflammatory pathways and immune suppression	May support CRC in chronic infections

**Table 3:** Beneficial bacteria—role in immune response and barrier integrity in CRC.

Beneficial Bacteria	Immune Response	Intestinal Barrier Integrity
*Lactobacillus* spp. [82,83]	- Stimulate anti-inflammatory cytokine production (e.g., IL-10, TGF-β). - Modulate dendritic cell maturation and T cell differentiation. - Suppress NF-κB signaling.	Increase tight junction proteins.
*Bifidobacterium* spp. [84,85]	- Promote immune tolerance by Tregs T cells. - Inhibit pro-inflammatory cytokines such as TNF-α and IL-6. - Reduce pathogenic bacteria colonization and their immune evasion.	Sustain intestinal barrier integrity by the production of SCFAs, especially acetate.
*Faecalibacterium prausnitzii* [86,87]	- Produces butyrate that inhibits inflammation and supports epithelial health. - Inhibits NF-κB activation, suppressing pro-inflammatory cytokine release. - Increase levels of IL-10 and reduce intestinal inflammation.	Influences the expression of tight junction proteins (such as occludin, claudin, and ZO-1) to reduce intestinal permeability.
*Akkermansia muciniphila* [88,89]	- Enhances the production of SCFAs and supports anti-inflammatory responses.	Strengthens the mucus layer, improving intestinal barrier function.
*Roseburia* spp. [90]	- Induce Treg differentiation and anti-inflammatory effect by high production of butyrate.	Reduces gut permeability, preventing the systemic spread of microbial toxins.
Clostridium cluster XIVa and IV [91]	- Maintain immune tolerance. - Inhibits NF-κB signaling, reducing the production of pro-inflammatory cytokines like TNF-α, IL-6, and IL-1β.	Butyrate and other SCFAs serve as an energy source for colonocytes, promoting epithelial cell proliferation and repair.
*Propionibacterium* spp. [92]	- Produce propionate, an SCFA that regulates immune cell activity and reduces inflammation. - Inhibit the growth of pathogenic bacteria.	Provides energy for colonic epithelial cells, supporting barrier integrity.
*Escherichia coli* (non-pathogenic strains) [93]	- Modulate the balance between pro-inflammatory and anti-inflammatory responses.	Enhance epithelial barrier function.
*Bacteroides fragilis* (Polysaccharide A-producing strains) [94]	- Polysaccharide A (PSA) stimulates Tregs and promotes anti-inflammatory cytokine production. - Reduces intestinal inflammation and promotes immune tolerance.	Maintain epithelial integrity.
*Enterococcus faecium* (Probiotic Strains) [88]	- Produce bacteriocins to inhibit pathogenic bacteria. - Support T cell activation and anti-inflammatory pathways.	Strengthening the intestinal barrier (enhancing tight junctions, mucus secretion, and SCFA production).
*Streptococcus thermophilus* [95]	- Produces bioactive peptides that enhance mucosal immunity. - Supports the production of SCFAs, aiding in immune regulation.	Restore epithelial cell structure, reducing inflammation-driven barrier damage. Increasing mucin production for better epithelial protection.

## Data Availability

No new data were created or analyzed in this study.
